# Cross-National Time Trends in Adolescent Mental Well-Being From 2002 to 2018 and the Explanatory Role of Schoolwork Pressure

**DOI:** 10.1016/j.jadohealth.2020.02.010

**Published:** 2020-06

**Authors:** Alina Cosma, Gonneke Stevens, Gina Martin, Elisa L. Duinhof, Sophie D. Walsh, Irene Garcia-Moya, András Költő, Inese Gobina, Natale Canale, Carolina Catunda, Jo Inchley, Margaretha de Looze

**Affiliations:** a Department of Interdisciplinary Social Science, Utrecht University, Utrecht, the Netherlands; b Faculty of Electrical Engineering and Computer Science, VSB – Technical University of Ostrava, Ostrava, Czech Republic; c Sts Cyril and Methodius Faculty of Theology, Olomouc University Social Health Institute, Palacky University in Olomouc, Olomouc, Czech Republic; d Human Environments Analysis Laboratory, Department of Geography, Western University, London, Canada; e Department of Criminology, Bar Ilan University, Ramat Gan, Israel; f Department of Developmental and Educational Psychology, University of Seville, Seville, Spain; g Health Promotion Research Centre, School of Health Sciences, National University of Ireland Galway, Galway, Ireland; h Department of Public Health and Epidemiology, Institute of Public Health, Riga Stradiņš University, Riga, Latvia; i Department of Developmental and Social Psychology, University of Padova, Padova, Italy; j Faculty of Language and Literature, Humanities, Arts and Education, Luxembourg University, Luxembourg; k MRC/CSO Social and Public Health Sciences Unit, University of Glasgow, Glasgow, United Kingdom; l School of Medicine, University of St Andrews, St Andrews, United Kingdom

**Keywords:** Mental health, Mental well-being, Well-being, Adolescent, Adolescence, Trends, Schoolwork pressure, Life satisfaction, Psychosomatic health complaints, Country variation, Cross-national, Multilevel analysis, HBSC

## Abstract

**Purpose:**

Previous research has shown inconsistent time trends in adolescent mental well-being, but potential underlying mechanisms for such trends are yet to be examined. This study investigates cross-national time trends in adolescent mental well-being (psychosomatic health complaints and life satisfaction) in mainly European countries and the extent to which time trends in schoolwork pressure explain these trends.

**Methods:**

Data from 915,054 adolescents from 36 countries (50.8% girls; mean_age_ = 13.54; standard deviation_age_ = 1.63) across five Health Behaviour in School-aged Children surveys (2002, 2006, 2010, 2014, and 2018) were included in the analyses. Hierarchical multilevel models estimated cross-national trends in adolescent mental well-being and schoolwork pressure. We also tested whether schoolwork pressure could explain these trends in mental well-being.

**Results:**

A small linear increase over time in psychosomatic complaints and schoolwork pressure was found. No change in life satisfaction emerged. Furthermore, there was large cross-country variation in the prevalence of, and trends over time in, adolescent mental well-being and schoolwork pressure. Overall, declines in well-being and increases in schoolwork pressure were apparent in the higher income countries. Across countries, the small increase in schoolwork pressure over time partly explained the increase in psychosomatic health complaints.

**Conclusions:**

Our findings do not provide evidence for substantial declines in mental well-being among adolescents. Yet, the small increase in mental well-being and increases in schoolwork pressure appear to be quite consistent across high-income countries. This calls for the attention of public health professionals and policy-makers. Country differences in trends in both adolescent mental well-being outcomes and schoolwork pressure were considerable, which requires caution regarding the cross-national generalization of national trends.

**Implications and Contribution**

Time trends in adolescent mental well-being diverged: a small but significant increase in psychosomatic complaints and a stable pattern in life satisfaction was observed. Trends in adolescent mental well-being and schoolwork pressure varied between countries. Changes in school pressure over time partly explained the decline in psychosomatic health complaints.

There is widespread societal concern about reported declines in adolescent mental well-being during the last two decades [1,2]. Several studies have concluded that adolescent mental well-being has deteriorated significantly since the beginning of the 21st century, especially in countries such as the U.S., the United Kingdom, Finland, Norway, and Sweden [[1], [2], [3], [4], [5], [6], [7], [8], [9]]. Yet, research in this field relies heavily on data from only a few mainly Western countries and studies conducted in different periods. Therefore, the extent to which the observed declines in adolescent mental well-being are consistent across countries is largely unknown. Moreover, little is known about the processes that could explain these declines in adolescent mental well-being [2]. One explanatory variable could be the experience of schoolwork pressure during these highly formative years [3], especially given its systematic association with adolescent mental health problems [10], and an increase in schoolwork pressure has been observed in some countries [11]. The present study presents trends in adolescent mental well-being between 2002 and 2018, across 36 European and North American countries and regions. In addition, it examines whether the observed trends in mental well-being can be explained by changes in schoolwork pressure over the same period.

Despite mental health and well-being becoming an increasing public health priority in many Western countries, evidence for a decline in mental well-being among adolescents remains mixed. For example, increases over time in mental health problems (especially emotional symptoms) have been reported in Finland [5], England [3], Norway [7], the U.S. [8], Scotland [11], and Sweden [4], with all these studies reporting stronger declines for girls than boys. Conversely, other studies have found relatively stable trends in adolescent mental health in Canada [12], the Netherlands [13], and Norway [9]. Furthermore, among the few studies investigating cross-country effects, some studies revealed different trends in mental well-being across countries [14,15], whereas others did not report such cross-country variations and report relatively stable trends from the 2000s onward [6]. Fundamental differences in the design, methodology (i.e., measurements), and period between these studies make it hard to compare their findings. Although most studies focused on the period before 2012, some more recent research [8] reported an especially steep decline in adolescent mental well-being after 2012. Using nationally representative samples of adolescents from 36 countries and employing standardized sampling and data collection methods across a 16-year time span, the present study offers a unique opportunity to explore cross-country variation in time trends in adolescent mental well-being.

Since the end of the 20th century, a continuous rise in perfectionism (e.g., higher expectations to perform well in school and university) has been observed among young people in the U.S., Canada, and the United Kingdom [16]. These heightened levels of perfectionism may have contributed to increases in adolescents' schoolwork pressure [17]. A cross-national comparative study in Europe and North America found that schoolwork pressure did not change systematically across countries between 1994 and 2010 [17]. However, in more recent studies, an increase in perceived schoolwork pressure has been reported in some European countries, such as Sweden [18] and the Czech Republic [19]. The transactional model of stress and coping [20] conceptualizes schoolwork pressure as the result of perceived imbalance between school demands and resources to meet them [21]. In line with this, high levels of schoolwork pressure have been found to be associated with poorer mental health among adolescents [10,[21], [22], [23]]. Schoolwork can be understood as a form of stress and may impact adolescent mental well-being in multiple pathways such as neurobiological processes [24], emotional self-regulation [25], or sense of control [26]. Thus, potential increases in perceived schoolwork pressure may explain at least a part of the expected decline in adolescent mental well-being in the last two decades.

Previous literature reported consistent gender, age, and socioeconomic differences in adolescent mental well-being. Time trend analyses showed that, compared with boys, girls (especially older adolescent girls) increasingly report more emotional problems [3], internalizing problems [1], lower life satisfaction [27], and more frequent multiple health complaints [28]. In addition, socioeconomic status has an impact on adolescent mental health [29]. Adolescents from socioeconomically disadvantaged groups report higher rates of poor subjective health [30], lower life satisfaction [27], more frequent psychosomatic health symptoms [31], and lower quality of life and well-being [32] than their peers from more advantaged families. Given all these associations, gender, age, and family affluence were included as control variables in the present study.

In sum, although many studies have reported recent declines in adolescent mental well-being, the literature is limited in terms of the comparability of periods examined, methods used, countries studied, and outcomes measured, and therefore it is difficult to compare across existing studies. The present study addresses these challenges by using data collected during the same school years (2001/2002, 2005/2006, 2009/2010, 2013/2014, and 2017/2018) and using standardized research protocols across 36 countries and regions. Schoolwork pressure has been suggested as a possible driver of change over time in adolescent mental well-being [3], but to the best of our knowledge, this has not yet been comprehensively tested. This study, therefore, addresses three research questions, using data from the international Health Behaviour in School-aged Children (HBSC) survey:

(1) To what extent are declines in two indicators of adolescent mental well-being (psychosomatic complaints and life satisfaction) observed between 2002 and 2018, across 36 European and North American countries and regions, and are there cross-national variations in these trends over time?
(2) To what extent are increases in schoolwork pressure observed between 2002 and 2018, across 36 European and North American countries and regions, and are there cross-national variations in this over time?
(3) To what extent do changes in schoolwork pressure explain trends over time in adolescent mental well-being?

## Methods

### Sample

The HBSC is a World Health Organization collaborative cross-national study that has been conducted every 4 years since 1983/1984 to monitor the health and well-being of adolescents across Europe and North America, using a standardized research protocol [33]. For each survey round, countries collected data from a nationally representative sample of 11-, 13-, and 15-year-olds. Stratified random cluster sampling was used with classes within schools as the primary sampling unit. Adolescents completed anonymous questionnaires in classroom settings. Questionnaires were translated from English into national languages with back-translation checks, following a validated protocol [33].

For this study, data from five rounds were used, covering the period between 2002 and 2018 (survey years: 2002, 2006, 2010, 2014, and 2018). Participating countries were eligible for the present analyses if they had collected data on psychosomatic complaints, life satisfaction, and schoolwork pressure in four of the five HBSC surveys included. This involved analyzing data from a total of 915,054 adolescents from 36 countries or regions (50.8% girls; mean_age_ = 13.54; standard deviation [*SD*]_age_ = 1.63). Institutional ethical consent was obtained in each participating country. Table 1 provides detailed description of the study sample.

**Table 1:** Description of the study sample (unweighted)

Variable	n (%)
Total	915,054
Number of countries/regions	36
Survey round	
2002	149,639 (16.4)
2006	184,463 (20.2)
2010	194,689 (21.3)
2014	193,205 (21.1)
2018	193,058 (21.1)
Gender	
Boys	450,329 (49.2)
Girls	464,725 (50.8)
Age group	
11-year-olds	298,601 (32.6)
13-year-olds	312,701 (34.2)
15-year-olds	296,716 (32.4)
Mean psychosomatic complaints (SD)^a^	8.08 (6.47)
Mean life satisfaction (SD)^b^	7.60 (1.91)
Mean schoolwork pressure (SD)^c^	1.26 (.90)

SD = standard deviation.

a Scale range: 0–32.

b Scale range: 0–10.

c Scale range: 0–3.

### Instruments

#### Adolescent mental well-being

Adolescent mental well-being was measured by two instruments: psychosomatic health complaints and life satisfaction. These measures tap into different complementary facets of mental well-being: internalizing symptoms (psychosomatic health complaints) and subjective well-being (life satisfaction).

#### Psychosomatic health complaints

Participants indicated the frequency with which they had experienced the following eight health complaints over the past 6 months: feeling low, irritability or bad temper, feeling nervous, difficulties in getting to sleep, feeling dizzy, headache, stomach ache, and backache, with the following response options: (1) “about every day,” (2) “more than once a week,” (3) “about every week,” (4) “about every month,” and (5) “rarely or never.” This instrument has adequate test–retest reliability and validity [34]. In this study, the aggregated Cronbach's alpha coefficient across all survey cycles was .81, and for individual survey cycles, it varied from .78 to .83, indicating a good internal consistency. Confirmatory factor analysis using all data confirmed that a one-factor structure showed a good fit (Confirmatory Fit Index = .923; Root Mean Square Error of Approximation = .086; and Tucker-Lewis Index = .893; *p* < .05). Responses were reversely recoded (0–4) so that higher scores indicated more psychosomatic complaints. Given the low missing data rates per individual health complaint (range per symptom between 1.7% and 2.9%), a sum score was created for those with no missing items on this scale (range 0–32).

#### Life satisfaction

Life satisfaction was assessed with the Cantril ladder [35]. Participants rated their life satisfaction on a scale ranging from (0) “the worst possible life” to (10) “the best possible life.” The Cantril ladder has been shown to be a reliable and valid instrument to measure overall mental well-being among adolescents [36,37].

#### Schoolwork pressure

Participants responded to the question, “How pressured do you feel by the schoolwork you have to do?” The response options available were “not at all” (1), “a little” (2), “some” (3), and “a lot” (4). The responses were recoded from 0 to 3, a higher score indicating more perceived pressure.

#### Gender and age

Participants were asked to indicate whether they are a boy or a girl, as well their date of their birth.

#### Family affluence

Socioeconomic status was measured using the Family Affluence Scale [38], which included items on the households' number of cars and computers, whether adolescents have their own bedroom, and the number of holidays spent abroad. Sum-scores were transformed into proportional ranks that indicate adolescents' relative family affluence in their residential country. This allowed to identify groups of young people in the lowest 20% (low affluence), middle 60% (medium affluence), and highest 20% (high affluence) within each country and region.

### Analytic strategy

For the three outcomes (psychosomatic health complaints, life satisfaction, and schoolwork pressure), hierarchical multilevel regression models were performed. To test the time trends in mental well-being and schoolwork pressure, the first model included gender, age, family affluence, and survey year (as a continuous variable) on the individual level, with psychosomatic health complaints and life satisfaction as outcomes. The second model included country on the second level. In a third model, an additional level for country/year was added to investigate the extent to which time trends in mental well-being varied between countries. Finally, for both mental well-being outcomes, a fourth model was run that included school pressure to examine the explanatory role of schoolwork pressure. In addition, Sobel's test was conducted to test for a mediation effect of schoolwork pressure on mental well-being. Although there are several approaches that can be used to investigate mediation, Sobel's test is considered a conservative approach, and given the large sample size and results of the hierarchical models, this conservative approach lends further evidence to the results of the hierarchical models. In addition, to describe country-specific time trends between 2002 and 2018 in adolescent psychosomatic health complaints, life satisfaction, and schoolwork pressure, we tested linear regression models controlled for gender, age, and family affluence.

Only individuals with complete data on each outcome were included in each set of hierarchical multilevel models. Multilevel models were conducted using the *runmlwin* command [39] via Stata v14 (StataCorp, College Station, TX) and MLwiN v3.03 (Centre for Multilevel Modelling, University of Bristol, Bristol, United Kingdom), with estimation procedures obtained by Markov Chain Monte Carlo method. Because no previous knowledge was assumed, diffuse prior distributions were used for all estimates. Initial values were derived from a least squares algorithm [39]. Bayesian Deviance Information Criterion was used to test for improvement of model fit, with lower values indicating better fit. Generally, a difference of five is considered a substantial improvement [40,41].

## Results

### Overall trends in adolescent mental well-being

Table 2 shows the overall trends in adolescent mental well-being. A small linear increase over time in psychosomatic health complaints (*B* = .067; *p* < .001; Model 1) was found. The overall mean in psychosomatic health complaints ranged from 7.74 (SD = 6.13) in 2002 to 8.67 (SD = 6.06) in 2018 (Supplementary Table A1). Girls (*B* = 2.421; *p* < .001) and older adolescents (*B* = .567; *p* < .001) reported higher levels of psychosomatic health complaints. Adolescents from medium (*B* = −.609; *p* < .001) and high family affluence (*B* = −.601; *p* < .001) reported lower levels of psychosomatic health complaints compared with those from low affluent families. Furthermore, no significant change over time (*B* = .000; *p* < .001) in life satisfaction was observed (Model 5). Overall, means in life satisfaction ranged from 7.53 (SD = 1.91) in 2002 to 7.69 (SD = 1.87) in 2018 (Supplementary Table A2). Girls (*B* = −.247; *p* < .001) and older adolescents (*B* = −.206; *p* < .001) reported lower levels of life satisfaction. Adolescents from medium (*B* = .420; *p* < .001) and high family affluence (*B* = .686; *p* < .001) reported higher levels of life satisfaction. By adding the country level (Models 2 and 6) and country/year level (Models 3 and 7), the model fit substantially improved compared with the initial Models 1 and 5 (more specifically, the Bayesian Deviance Information Criterion declined when the country and country-year level was included). This indicates that there is significant unexplained variance across countries and over time within countries [34].

**Table 2:** The results of multilevel linear regression predicting time trends in adolescent mental health in 36 countries and the explanatory role of schoolwork pressure

	Psychosomatic complaints (n = 824,998)				Life satisfaction (n = 829,339)			
	*B* (95% CI)	*B* (95% CI)	*B* (95% CI)	*B* (95% CI)	*B* (95% CI)	*B* (95% CI)	*B* (95% CI)	*B* (95% CI)
	Model 1	Model 2	Model 3	Model 4	Model 5	Model 6	Model 7	Model 8
Fixed effects								
Gender (Boys = ref)	2.421 (2.394, 2.448)∗∗∗	2.421 (2.395, 2.448)∗∗∗	2.421 (2.394, 2.447)∗∗∗	2.228 (2.202, 2.254)∗∗∗	−.247 (−.255, −.239)∗∗∗	−.245 (−.253, −.237)∗∗∗	−.245 (−.253, −.237) ∗∗∗	−.206 (−.214, −.200)∗∗∗
Age	.567 (.558, .575)∗∗∗	.564 (.556, .572)∗∗∗	.567 (.559, .575)∗∗∗	.362 (.354, .370)∗∗∗	−.206 (−.208, −.203)∗∗∗	−.204 (−.207, −.202)∗∗∗	−.203 (−.206 −.201) ∗∗∗	−.160 (−.163, −.158)∗∗∗
Medium FAS (low FAS = ref)	−.609 (−.644, −.574)∗∗∗	−575 (−.609, −.540)∗∗∗	−.573 (−.608, −.538)∗∗∗	−.556 (−.589, −.522)∗∗∗	.420 (.410, .431)∗∗∗	.420 (.410, .430)∗∗∗	.425 (.415, .436)∗∗∗	.422 (.412, .432)∗∗∗
High FAS (low FAS = ref)	−.601 (−.645, −.558)∗∗∗	−.571 (−.614, −.528)∗∗∗	−.573 (−.616, −.529)∗∗∗	−.581 (−.623, −.539)∗∗∗	.686 (.673, .699)∗∗∗	.704 (.691, .716)∗∗∗	.709 (.696, .722)∗∗∗	.711 (.699, .724)∗∗∗
Survey year	.067 (.064, .069)∗∗∗	.058 (.055, .060)∗∗∗	.061 (.046, .077)∗∗∗	.054 (.039, .068)∗∗∗	.000 (.000, .001)	.001 (.000, .001)	.000 (−.005, .005)	.001 (−.003, .006)
Schoolwork pressure				1.981 (1.966, 1.996)∗∗∗				−.414 (−.419, −.410)∗∗∗
Random effects								
Student variance	39.047 (38.928, 39.167)	38.175 (38.058, 38.2912)	37.962 (.37.846, 38.079)	35.073 (34.966 35.180)	3.426 (3.415, 3.44)	3.386 (3.376, 3.396)	3.362 (3.451, 3.372)	3.236 (3.225, 3.245)
Country variance		1.031 (.637, 1.662)	1.00 (.593, 1.652)	1.079 (.643, 1.77)		.043 (.027, .069)	.037 (.020, .064)	.048 (.027, .082)
Country/year variance			.303(.238, .387)	.277 (.216, .353)			.033 (.026, .042)	.034 (.026, .043)
Bayesian DIC	5364678.98	5346083.05	5341600.74	5276287.26	3374824.90	3365156.52	3359277.92	3327560.40

CI = credible interval; DIC = Deviance Information Criterion; FAS = Family Affluence Scale.

∗∗∗*p* < .001; ∗∗*p* < .01.

### Cross-country variation in the trends in adolescent mental well-being

Table 3 presents the linear trends in psychosomatic health complaints, life satisfaction, and schoolwork pressure by country. Overall, the results show small linear changes between 2002 and 2018. Across most countries (26 of 36), a significant increase in psychosomatic health complaints was observed, with some countries (particularly Greenland, *B* = .182; *p* < .001) showing relatively stronger year-by-year increases than others (for instance, Canada, *B* = .031; *p* < .001). A significant decrease over time was found in Slovakia, Spain, and Ukraine, whereas no linear change was observed in Croatia, Czech Republic, England, Greece, Lithuania, and Norway (Supplementary Table A1 provides mean scores across each survey cycle).

**Table 3:** Linear changes over time within countries in adolescent mental health and schoolwork pressure (2002–2018)

	Psychosomatic complaints^c^		Change per year	Life satisfaction^d^		Change per year	Schoolwork pressure^e^		Change per year
	2002^a^	Difference 2002 − 2018	*B* (±95% CI)	2002^a^	Difference 2002 − 2018	*B* (±95% CI)	2002^a^	Difference 2002 − 2018	*B* (±95% CI)
Austria	6.13	1.82	.111∗∗∗	7.95	−.24	−.011∗∗∗	1.07	.02	−.002∗∗
Belgium Flemish	6.55	1.38	.100∗∗∗	7.72	.08	−.022∗∗∗	1.12	−.03	.001
Belgium French	8.02	1.35	.108∗∗∗	−	−	−	.97	.30	.022∗∗∗
Canada	8.09	.73	.031∗∗∗	7.56	−.30	−.015∗∗∗	1.31	.18	.007∗∗∗
Croatia	6.98	.30	.004	7.49	.60	.045∗∗∗	1.13	.21	.009∗∗∗
Czech Republic	8.17	.28	−.060	7.45	.33	.011∗∗∗	1.08	.24	.012∗∗∗
Denmark	6.93	.87	.077∗∗∗	7.72	−.04	−.014∗∗∗	1.08	.00	.003∗
England	9.69	−.31	−.015	7.27	.17	−.008∗∗∗	1.54	.05	.001
Estonia	8.08	1.21	.084∗∗∗	7.17	.56	.016∗∗∗	1.44	.01	.002
Finland	8.5	1.27	.071∗∗∗	7.95	−.12	−.020∗∗∗	1.38	.12	.006∗∗∗
France	8.27	1.23	.075∗∗∗	7.58	.08	.001	1.02	.02	.001
Germany	6.44	1.75	.101∗∗∗	7.53	.16	.002	1.10	.03	.004∗∗∗
Greece	8.78	.46	−.007	7.99	−.45	−.035∗∗∗	1.33	−.15	−.012∗∗∗
Greenland	6.23	2.33	.182∗∗∗	7.47	.41	.002	.96	−.11	.002
Hungary	8.69	1.02	.058∗∗∗	7.55	.03	.002	1.10	.00	−.003∗∗
Iceland^b^	8.55	1.00	.060∗∗∗	7.80	−.18	−.006∗∗∗	1.33	.27	.019∗∗∗
Ireland	6.99	1.33	.113∗∗∗	7.61	−.06	−.016∗∗	1.23	.15	.011∗∗∗
Italy	10.28	1.08	.067∗∗∗	7.43	.15	.001	1.43	.28	.016∗∗∗
Latvia	7.37	1.66	.102∗∗∗	7.01	.39	.015∗∗∗	1.22	−.14	−.007∗∗∗
Lithuania	7.73	.17	.020	7.06	.85	.038∗∗∗	1.69	.07	−.02
Luxembourg	8.71	.92	.108∗∗∗	7.49	.15	−.004	1.23	.06	.007∗∗∗
Netherlands	6.34	.92	.109∗∗∗	8.14	−.02	−.025∗∗∗	.84	.35	.019∗∗∗
North Macedonia	6.45	1.94	.090∗∗∗	8.45	−.36	−.020∗∗∗	1.29	.19	.016∗∗∗
Norway	7.29	.19	.018	7.45	.45	.016∗∗∗	1.27	.05	.003∗∗
Poland	7.91	1.24	.092∗∗∗	7.35	.13	−.004	1.46	−.05	−.010∗∗∗
Portugal	6.49	.80	.100∗∗∗	7.40	.33	.010∗∗∗	1.42	−.09	−.007∗∗∗
Romania^b^	9.63	−.65	−.020	7.72	.61	.048∗∗∗	1.28	.11	.007∗∗∗
Russia	6.73	1.16	.072∗∗∗	7.10	.31	.002	1.07	−.19	−.008∗∗∗
Scotland	7.75	.77	.072∗∗∗	7.66	−.04	−.002	1.24	.16	.014∗∗∗
Slovakia^b^	9.63	−.61	−.026∗∗∗	7.80	−.16	−.026∗∗∗	1.25	−.25	−.015∗∗∗
Slovenia	6.41	.85	.086∗∗∗	7.66	.31	.013∗∗∗	1.49	.09	.001
Spain	8.25	−1.81	−.087∗∗∗	7.68	.41	.014∗∗∗	1.50	.20	.012∗∗∗
Sweden	9.52	1.11	.077∗∗∗	7.59	−.13	−.022∗∗∗	1.24	.18	.005∗∗∗
Switzerland	7.78	.83	.053∗∗∗	7.82	−.13	−.019∗∗∗	1.01	.09	.007∗∗∗
Ukraine	9.24	−.04	−.230∗∗	6.97	.72	.034∗∗∗	1.01	−.09	−.014∗∗∗
Wales	8.17	.79	.088∗∗∗	7.37	.23	.009∗∗∗	1.55	−.03	−.005∗∗∗

CI = confidence interval.

∗∗∗*p* < .001; ∗∗*p* < .01, ∗*p* < .05.

a Scale: 0–32.

b Scale: 0–10.

c Scale: 0–3.

d Mean estimates.

e First survey in 2006. Adjusted for age, gender, and family affluence. No data available for French Belgium.

In contrast, linear increases in life satisfaction were found in one third of the countries (13 of 35), with Romania, Croatia Lithuania, and Ukraine showing relatively high year-on-year increases. A decrease in life satisfaction was observed in 13 countries (particularly in Greece, *B* = −.035; *p* < .001), whereas no changes in life satisfaction occurred in 10 countries. Consistent trends in both psychosomatic complaints and life satisfaction were found in only 12 countries. Overall declines in mental well-being (increasing psychosomatic complaints and declining life satisfaction) were observed in Austria, Belgium (Flemish), Canada, Denmark, Finland, Ireland, the Netherlands, North Macedonia, Sweden, and Switzerland. In contrast, improvements in mental well-being (declines in psychosomatic health complaints and increases in life satisfaction) were observed in Spain and Ukraine (Supplementary Table A1 and A2).

### Overall and cross-country trends in schoolwork pressure

The overall means in schoolwork pressure ranged from 1.22 (SD = .87) in 2002 to 1.32 (SD = .93) in 2018 (Supplementary Table A3). In the adjusted models (Table 4), an overall small increase in schoolwork pressure across all countries was observed (*B* = .004; *p* = .001), with girls (*B* = .092; *p* < .001) and older adolescents (*B* = .107; *p* < .001) reporting higher levels of schoolwork pressure. In addition, lower levels of schoolwork pressure were found among adolescents with a medium compared with low family affluence. Time trends in schoolwork pressure differed between countries (Model 3, Table 4). In line with this, increases in schoolwork pressure over time were observed in 20 of 36 countries (greatest increases were found in French Belgium, Iceland, the Netherlands, and North Macedonia), whereas decreases emerged in seven countries (greatest decreases were found in Greece and Ukraine). No change was found in nine countries (Supplementary Table A3).

**Table 4:** The results of multilevel linear regression predicting trends in schoolwork pressure

	Schoolwork pressure (n = 853,458)		
	*B* (95% CI)	*B* (95% CI)	*B* (95% CI)
	Model 1	Model 2	Model 3
Fixed effects			
Gender (boys = ref)	.092 (.089, 096)∗∗∗	.095 (.091, .099)∗∗∗	.095 (.091, .099)∗∗∗
Age	.107 (.106, 108)∗∗∗	.103 (.102, .105)∗∗∗	.103 (.102, .105)∗∗∗
Medium FAS (low FAS = ref)	−.008 (−.013, −.003)∗∗	−.011 (−.016, −.006)∗∗∗	−.008 (−.012, −.003)∗∗
High FAS (low FAS = ref)	−.005 (−.011, 001)	.000 (−.006, .000)	.007 (.001, .013)
Survey year	.004 (.004, .005)∗∗∗	.004 (.004, .004)∗∗∗	.004 (.001, .006)
Random effects			
Student variance	.776 (.773, .778)	.744 (.742, .746)	.737 (.784, .734)
Country variance		.037 (.023, .059)	.035 (.021, .058)
Country/year variance			.010 (.008, .013)
Bayesian DIC	2205355.29	2169434.11	2161297.12

CI = credible interval; DIC = Deviance Information Criterion; FAS = Family Affluence Scale.

∗∗∗ < .001; ∗∗*p* < .01.

### The explanatory role of schoolwork pressure in the time trends in adolescent mental well-being

Schoolwork pressure was associated with more frequent psychosomatic health complaints and lower life satisfaction (Models 4 and 8, Table 2). When schoolwork pressure was added to the models, the model fit improved substantially across both mental well-being indicators. In addition, the effect of survey year on psychosomatic health complaints slightly reduced but remained significant (Model 4). By adding schoolwork pressure to the model, the effect of survey year on life satisfaction remained nonsignificant (Model 8). The Sobel's test demonstrated that adding schoolwork pressure to the model significantly decreased time trends in psychosomatic health complaints (*z* = 2.72; *p* = .007). This means that across countries, the increase in schoolwork pressure partly explained the increase in psychosomatic health complaints.

## Discussion

The present study investigated time trends in adolescent mental well-being between 2002 and 2018 across 36 European and North American countries and regions. Overall, we found a small but significant increase in psychosomatic health complaints and no overall change in life satisfaction. Based on these two indicators, our findings do not provide evidence of a dramatic decline in adolescent mental well-being at a population level [8,42], as the effect size was rather small, and the different time trends trajectories for these two indicators of mental well-being suggest a more complex pattern. Over the same period, a small overall increase in perceived schoolwork pressure was also observed, and this explained a very small proportion of the increase in psychosomatic health complaints. However, country differences in trends in both adolescent mental well-being and perceived schoolwork pressure were considerable, which highlights the need for caution regarding the cross-national generalization of national trends. Furthermore, the outcomes varied by age, gender, and family affluence with girls, older adolescents, and those from lower family affluence backgrounds showing poorer mental well-being profiles at each time point.

The different trends in psychosomatic complaints and life satisfaction reinforce the idea that mental well-being is a multidimensional construct and that different components of mental well-being can show different trajectories and may have differential susceptibilities. Life satisfaction, which refers to global cognitive evaluations about one's life, can be considered a global construct of subjective well-being [37] and may, therefore, be influenced by broader life experiences and relationships. In contrast, psychosomatic complaints may represent symptoms of more immediate stress, which, at the more severe end, may impair everyday functioning [35]. Together, our findings indicate that although, on average, adolescents have not become less satisfied with their lives, nowadays, they do experience slightly more psychosomatic health complaints and schoolwork pressure than 16 years ago. Similarly, affective or emotional aspects of well-being have been considered to be more susceptible to fluctuations compared with life satisfaction, which is usually described as a more stable component [43]. These findings strengthen a multidimensional view of mental well-being [44] and reflect a need for greater understanding of the associations between risk factors and different aspects of mental well-being.

Beyond the general pattern, considerable country differences were observed. First, countries, in which one or both indicators of well-being reflected an improvement, were mainly Eastern European (i.e., Czech Republic, Romania, and Ukraine). As such, our data may point to a recovery effect for adolescents in Eastern Europe, whose mental health and well-being levels were typically lower than those of adolescents in Western Europe until the late 20th century [44]. Among adults, the transition of Central and Eastern European countries from communism to capitalism in the 1990s was reflected by a decrease and then a recovery in life satisfaction [45]. Our data may reflect a similar development among adolescents [46]. There is therefore a need to better understand the country-specific processes and mechanisms, which may affect adolescent mental well-being particularly in relation to the economic, cultural, educational, and social circumstances in which young people are growing up. What factors distinguish countries with improvement in adolescent mental well-being from those with declines over time? This scientific knowledge is of great importance to the development of country group–specific interventions tailored to strengthen adolescent mental health throughout Europe.

In contrast, a “double decline” in mental well-being was observed in 10 countries whereby both psychosomatic symptoms and life satisfaction showed a worsening trend. Still, it must be added that these countries were not necessarily among those with the lowest mental well-being in 2017/2018: two of these countries scored beneath the general average on life satisfaction, whereas four of them scored above the mean on psychosomatic symptoms. These countries are all relatively wealthy and gender equal (i.e., Austria, Flemish Belgium, Canada, Denmark, Finland, Iceland, Ireland, the Netherlands, Sweden, and Switzerland), and these country-level characteristics have been indicated earlier as potentially protective for adolescent mental well-being [27,47]. Accordingly, these countries mostly scored below European average on psychosomatic health complaints and above average on life satisfaction in 2002 [44]. Future research should examine in more detail why trends over time in adolescent mental well-being are worsening in this specific group of countries.

Although overall perceived schoolwork pressure increased over time, this trend only explained a very small part of the overall time trends in adolescent mental well-being. Our findings suggest that schoolwork pressure partially mediates the increase in psychosomatic health complaints. Future research needs to investigate the role of other factors that could contribute to explaining the trends in adolescent mental well-being, such as increased use of social media [8] or changes in the quality of family and peer relationships [2].

In line with previous research [1,3,28], our study indicates that girls show a considerably higher risk of both psychosomatic health complaints and low life satisfaction compared with boys. Possible mechanisms that could explain the lower mental well-being of girls include the fact that girls are expected to be more emotionally sensitive and expressive [47], experience more restricted gender roles and body dissatisfaction [14], are more likely to experience and communicate health symptoms [48], or experience more school performance pressure [10]. This makes them an important target for preventive interventions.

This study has several strengths such as the large, nationally representative samples of adolescents across 36 European and North American countries, the use of a standard protocol for data collection and the coverage of a wide and recent period (16 years). Limitations include the cross-sectional, self-report nature of the data. The measures used were restricted to those available in the HBSC study since 2002 and focus on specific elements of mental well-being. Particularly, our study would have benefitted from the inclusion of other mental well-being outcomes, such as anxiety, depression, behavioral, or conduct problems, as this would have provided us with a more complete picture of time trends in adolescent mental health. Further research that includes a wider range of mental health outcome measures and other potential drivers of adolescent mental health trends, such as social media use, are required to better understand this complex issue.

In conclusion, our study has several implications. The findings present a mixed picture across the 36 countries and regions included, with no evidence of universal trends. Although there were clear declines in mental well-being in a number of countries, particularly those in Northern and Western Europe and Canada, in other countries, favorable or more stable trends were observed. There is, therefore, a need to better understand the country-specific processes and mechanisms, which may affect adolescent mental well-being particularly in relation to the economic, cultural, educational, and social circumstances in which young people are growing up. What factors distinguish countries with improvement in adolescent mental well-being from those with declines over time? This scientific knowledge is of great importance to the development of country group specific interventions tailored to strengthen adolescent mental health throughout Europe. Education policies must pay attention to the deleterious impact of schoolwork pressure on mental well-being and seek to create supportive learning environments, which ensure that young people have the necessary resources to manage academic demands. In line with previous research [49], the results suggest that the stress resulting from high levels of schoolwork pressure may at least partially account for the excess in psychosomatic complaints observed among girls and older adolescents. School-based interventions to enhance coping skills and stress reduction strategies among these groups may, therefore, be particularly beneficial. Beyond the school environment, further studies should focus on other possible factors, which may explain country-level differences in adolescent mental health to inform actions to support healthy development across the adolescent years.

## Supplementary Data

Supplementary Tables A1–A3
