# Supplementary material for: Cross-National Time Trends in Adolescent Mental Well-Being From 2002 to 2018 and the Explanatory Role of Schoolwork Pressure
Source: J Adolesc Health. 2020 Jun;66(6 Suppl):S50–8. doi: 10.1016/j.jadohealth.2020.02.010 (PMC8131201; doi:10.1016/j.jadohealth.2020.02.010)
Supplement: Supplementary Tables A1–A3 [file mmc1.docx]

Supplementary Table A1. Time trends in psychosomatic health complaints by country

|  | 2002 | 2006 | 2010 | 2014 | 2018 | Change per year |
| --- | --- | --- | --- | --- | --- | --- |
|  | Mean | Mean | Mean | Mean | Mean | *B* (±95% CI) |
| Austria | 6.13 | 5.45 | 6.04 | 6.48 | 7.95 | .111(.091 , .131)*** |
| Belgium (Flemish) | 6.55 | 6.33 | 6.44 | 7.15 | 7.93 | .100(.082 , .118)*** |
| Belgium (French) | 8.02 | 8.55 | 8.55 | 9.10 | 9.37 | .108(.091 , .125)*** |
| Canada | 8.09 | 8.60 | 8.44 | 8.30 | 8.82 | .031(.015 , .047)*** |
| Croatia | 6.98 | 7.62 | 7.59 | 7.80 | 7.28 | .004(− .015 , .023) |
| Czech Republic | 8.17 | 8.97 | 9.48 | 8.35 | 8.45 | − .060(− .021 , .008) |
| Denmark | 6.93 | 6.63 | 6.75 | 7.80 | 7.80 | .077(.059 , .095)*** |
| England | 9.69 | 8.10 | 8.23 | 7.77 | 9.38 | − .015(− .037 , .008) |
| Estonia | 8.08 | 7.58 | 7.85 | 7.99 | 9.29 | .084(.062 , .107)*** |
| Finland | 8.50 | 8.60 | 8.51 | − | 9.77 | .071(.051 , .092)*** |
| France | 8.27 | 9.06 | 8.88 | 9.77 | 9.50 | .075(.063 , .087)*** |
| Germany | 6.44 | 7.03 | 6.85 | 7.40 | 8.19 | .101(.079 , .123)*** |
| Greece | 8.78 | 8.36 | 8.06 | 7.65 | 9.24 | − .007(− .027 , .012) |
| Greenland | 6.23 | 5.73 | 6.16 | 7.06 | 8.56 | .182(.128 , .235)*** |
| Hungary | 8.69 | 9.04 | 8.71 | 9.07 | 9.71 | .058(.037 , .080)*** |
| Iceland | − | 8.55 | 8.69 | 9.37 | 9.55 | .060(.037 , .082)*** |
| Ireland | 6.99 | 6.90 | 7.57 | 8.05 | 8.32 | .113(.092 , .134)*** |
| Italy | 10.28 | 10.12 | 9.99 | 10.47 | 11.36 | .067(.049 , .086)*** |
| Latvia | 7.37 | 8.45 | 8.14 | 8.63 | 9.03 | .102(.081 , .123)*** |
| Lithuania | 7.73 | 8.09 | 8.29 | 7.70 | 7.90 | .020(− .002 , .043) |
| Luxembourg | − | 8.71 | 8.18 | 9.63 | 9.63 | .108(.082 , .134)*** |
| Netherlands | 6.34 | 5.25 | 6.21 | 7.52 | 7.26 | .109(.092 , .127)*** |
| North Macedonia | 6.45 | 6.62 | 6.23 | 7.47 | 8.39 | .090(.069 , .111)*** |
| Norway | 7.29 | 7.25 | 7.78 | 7.09 | 7.48 | .018(− .001 , .036) |
| Poland | 7.91 | 8.13 | 8.41 | 8.94 | 9.15 | .092(.075 , .109)*** |
| Portugal | 6.49 | 5.34 | 5.63 | 5.62 | 7.29 | .100(.082 , .119)*** |
| Romania | − | 9.63 | 8.90 | 8.64 | 8.98 | − .020(− .049 , .009) |
| Russia | 6.73 | 7.79 | 7.47 | 7.99 | 7.89 | .072(.053 , .091)*** |
| Scotland | 7.75 | 7.06 | 7.63 | 7.89 | 8.52 | .072(.052 , .092)*** |
| Slovakia | − | 9.44 | 8.46 | 8.59 | 8.83 | − .026(− .070 , − .018)*** |
| Slovenia | 6.41 | 5.96 | 5.33 | 7.37 | 7.26 | .086(.069 , .104)*** |
| Spain | 8.25 | 6.92 | 7.06 | 6.87 | 6.44 | − .087(− .104 , − .070)*** |
| Sweden | 9.52 | 8.60 | 8.71 | 9.59 | 10.63 | .077(.056 , .098)*** |
| Switzerland | 7.78 | 8.43 | 8.30 | 8.89 | 8.61 | .053(.039 , .067)*** |
| Ukraine | 9.24 | 9.43 | 8.76 | 7.65 | 9.20 | − .230(− .041 , − .006)** |
| Wales | 8.17 | 7.89 | 7.43 | 8.18 | 8.96 | .088(.068 , .108)*** |
| Total | 7.74 | 7.79 | 7.91 | 8.19 | 8.67 | *NA* |

^***^ *< .001;* ^**^ *< .01;* Adjusted for age and gender; − No data collected that survey year

Supplementary Table A2. Time trends in life satisfaction by country

|  | 2002 | 2006 | 2010 | 2014 | 2018 | Change per year |
| --- | --- | --- | --- | --- | --- | --- |
|  | Mean | Mean | Mean | Mean | Mean | *B* (±95% CI) |
| Austria | 7.95 | 7.83 | 7.58 | 7.95 | 7.71 | − .011(− .017 , − .005)*** |
| Belgium (Flemish) | 7.72 | 7.70 | 7.64 | 7.07 | 7.80 | − .022(− .027 , − .016)*** |
| Belgium (French) | − | − | 7.53 | 7.53 | 7.68 | − |
| Canada | 7.56 | 7.42 | 7.36 | 7.38 | 7.26 | − .015(− .019 , − .010)*** |
| Croatia | 7.49 | 7.34 | 7.53 | 7.91 | 8.09 | .045(.039 , .051)*** |
| Czech Republic | 7.45 | 7.28 | 7.51 | 7.20 | 7.78 | .011(.006 , .016)*** |
| Denmark | 7.72 | 7.82 | 7.53 | 7.63 | 7.68 | − .014(− .020 , .009)*** |
| England | 7.27 | 7.51 | 7.44 | 7.39 | 7.44 | − .008(− .013 , − .002)** |
| Estonia | 7.17 | 7.64 | 7.67 | 7.79 | 7.73 | .016(.009 , .022)*** |
| Finland | 7.95 | 7.94 | 7.77 | 7.69 | 7.83 | − .020(− .025 , − .016)*** |
| France | 7.58 | 7.48 | 7.53 | 7.35 | 7.66 | .001(− .004 , .004) |
| Germany | 7.53 | 7.33 | 7.40 | 7.38 | 7.69 | .002(− .013 , .016) |
| Greece | − | 7.99 | 7.85 | 7.72 | 7.54 | − .035(− .043 , − .027)*** |
| Greenland | 7.47 | 7.84 | 7.90 | 7.79 | 7.88 | .002(− .010 , .015) |
| Hungary | 7.55 | 7.26 | 7.40 | 7.54 | 7.58 | .002(− .004 , .009) |
| Iceland | − | 7.80 | 7.97 | 7.73 | 7.62 | − .006(− .013 , − .001) |
| Ireland | 7.61 | 7.72 | 7.60 | 7.59 | 7.55 | − .016(− .022 , − .010)*** |
| Italy | 7.43 | 7.52 | 7.53 | 7.36 | 7.58 | .001(− .005 , .006) |
| Latvia | 7.01 | 6.94 | 7.33 | 7.35 | 7.40 | .015(.009 , .021)*** |
| Lithuania | 7.06 | 7.31 | 7.56 | 7.85 | 7.91 | .038(.032 , .044)*** |
| Luxembourg | − | 7.49 | 7.65 | 7.44 | 7.64 | − .004(− .011 , .003) |
| Netherlands | 8.14 | 7.88 | 8.00 | 7.75 | 7.78 | − .025(− .030 , − .021)*** |
| North Macedonia | 8.45 | 8.20 | 8.13 | 7.66 | 8.43 | − .020(− .030 , − .009)*** |
| Norway | 7.45 | 7.87 | 7.78 | 7.89 | 7.9 | .016(.010 , .021)*** |
| Poland | 7.35 | 7.30 | 7.22 | 7.38 | 7.48 | − .004(− .010 , .001) |
| Portugal | 7.40 | 7.39 | 7.49 | 7.49 | 7.73 | .010(.004 , .015)*** |
| Romania | − | 7.72 | 7.57 | 8.12 | 8.33 | .048(.040 , .056)*** |
| Russia | 7.10 | 7.28 | 7.51 | 7.30 | 7.41 | .002(− .003 , .008) |
| Scotland | 7.66 | 7.45 | 7.65 | 7.74 | 7.62 | − .002(− .007 , .004) |
| Slovakia | − | 7.8 | 7.45 | 7.40 | 7.64 | − .026(− .040 , − .012)*** |
| Slovenia | 7.66 | 7.54 | 7.67 | 7.73 | 7.97 | .013(.007 , .018)*** |
| Spain | 7.68 | 7.99 | 7.96 | 7.85 | 8.09 | .014(.009 , .019)*** |
| Sweden | 7.59 | 7.81 | 7.72 | 7.36 | 7.46 | − .022(− .027 , − .016)*** |
| Switzerland | 7.82 | 7.78 | 7.70 | 7.73 | 7.69 | − .019(− .023 , − .015)*** |
| Ukraine | 6.97 | 7.23 | 7.22 | 7.65 | 7.69 | .034(.028 , .040)*** |
| Wales | 7.37 | 7.13 | 7.35 | 7.30 | 7.60 | .009(.004 , .014)*** |
| Total | 7.53 | 7.59 | 7.59 | 7.57 | 7.69 | *NA* |

^***^ *< .001;* ^**^ *< .01;* Adjusted for age and gender; − No data collected that survey year

Supplementary Table A3. Time trends in schoolwork pressure by country

| Country | 2002 | 2006 | 2010 | 2014 | 2018 | Change per year |
| --- | --- | --- | --- | --- | --- | --- |
|  | Mean | Mean | Mean | Mean | Mean | *B* (±95% CI) |
| Austria | 1.07 | 1.02 | 1.07 | .99 | 1.09 | − .002(− .005 , .001) |
| Belgium (Flemish) | 1.12 | 1.07 | 1.15 | 1.16 | 1.09 | .001(− .002 , .004) |
| Belgium (French) | .97 | 1.01 | .99 | 1.19 | 1.27 | .022(.019 , .024)*** |
| Canada | 1.31 | 1.43 | 1.39 | 1.36 | 1.49 | .007(.004 , .009)*** |
| Croatia | 1.13 | 1.13 | 1.18 | 1.22 | 1.34 | .009(.006 , .012)*** |
| Czech Republic | 1.08 | 1.23 | 1.18 | 1.24 | 1.32 | .012(.010 , .015)*** |
| Denmark | 1.08 | 1.18 | 1.18 | 1.20 | 1.08 | .003(.001 , .006)* |
| England | 1.54 | 1.62 | 1.45 | 1.44 | 1.59 | .001(− .003 , .004) |
| Estonia | 1.44 | 1.36 | 1.28 | 1.42 | 1.45 | .002(− .001 , .005) |
| Finland | 1.38 | 1.38 | 1.42 | 1.45 | 1.50 | .006(.004 , .009)*** |
| France | 1.02 | 1.03 | .95 | 1.00 | 1.04 | .001(− .001 , .002) |
| Germany | 1.10 | 1.11 | 1.10 | 1.18 | 1.13 | .004(.002 , .006)*** |
| Greece | 1.33 | 1.29 | 1.31 | 1.18 | 1.18 | − .012(− .015 , − .009)*** |
| Greenland | .96 | .84 | .85 | .99 | .85 | .002(− .003 , .007) |
| Hungary | 1.10 | 1.09 | .99 | .96 | 1.10 | − .003(− .006 , .001)* |
| Iceland | − | 1.33 | 1.38 | 1.51 | 1.60 | .019(.016 , .022)*** |
| Ireland | 1.23 | 1.31 | 1.29 | 1.40 | 1.38 | .011(.007 , .014)*** |
| Italy | 1.43 | 1.57 | 1.44 | 1.63 | 1.71 | .016(.013 , .019)*** |
| Latvia | 1.22 | 1.21 | .99 | 1.14 | 1.08 | − .007(− .009 , − .004)*** |
| Lithuania | 1.69 | 1.46 | 1.42 | 1.45 | 1.76 | − .002(− .007 , .004) |
| Luxembourg | − | 1.23 | 1.14 | 1.19 | 1.29 | .007(.004 , .011)*** |
| Netherlands | .84 | .92 | .91 | .99 | 1.19 | .019(.016 , .022)*** |
| North Macedonia | 1.29 | 1.15 | 1.27 | 1.45 | 1.48 | .016(.012 , .020)* |
| Norway | 1.27 | 1.29 | 1.24 | 1.20 | 1.32 | .003(.001 , .006)* |
| Poland | 1.46 | 1.55 | 1.07 | 1.24 | 1.41 | − .010(− .013 , − .007)*** |
| Portugal | 1.42 | 1.45 | 1.41 | 1.11 | 1.33 | − .007(− .010 , − .004)*** |
| Romania | − | 1.28 | 1.21 | 1.08 | 1.39 | .007(.002 , .011)*** |
| Russia | 1.07 | 1.12 | 1.09 | 1.00 | .88 | − .008(− .010 , − .006)*** |
| Scotland | 1.24 | 1.13 | 1.28 | 1.39 | 1.40 | .014(.011 , .018)*** |
| Slovakia | − | 1.25 | .90 | .97 | 1.00 | − .015(− .031 , .002) |
| Slovenia | 1.49 | 1.59 | 1.54 | 1.54 | 1.58 | .001(− .002 , .004) |
| Spain | 1.50 | 1.49 | 1.64 | 1.61 | 1.70 | .012(.009 , .014)*** |
| Sweden | 1.24 | 1.20 | 1.04 | 1.21 | 1.42 | .005(.002 , .008)*** |
| Switzerland | 1.01 | 1.05 | 1.11 | 1.13 | 1.10 | .007(.005 , .010)*** |
| Ukraine | 1.01 | 1.17 | .99 | .72 | .92 | − .014(− .017 , − .012)*** |
| Wales | 1.55 | 1.46 | 1.32 | 1.34 | 1.52 | − .005(− .008 , − .001)** |
| Austria | 1.07 | 1.02 | 1.07 | .99 | 1.09 | − .002(− .005 , .001) |
| Total | 1.24 | 1.26 | 1.22 | 1.26 | 1.32 | *NA* |

^***^ *< .001;* ^**^ *< .01;* Adjusted for age and gender; − No data collected that survey year
